# Vitamin C in the Presence of Sub-Inhibitory Concentration of Aminoglycosides and Fluoroquinolones Alters *Proteus mirabilis* Biofilm Inhibitory Rate

**DOI:** 10.3390/antibiotics8030116

**Published:** 2019-08-11

**Authors:** Joanna Kwiecińska-Piróg, Krzysztof Skowron, Tomasz Bogiel, Agata Białucha, Jana Przekwas, Eugenia Gospodarek-Komkowska

**Affiliations:** 1 Department of Microbiology, Nicolaus Copernicus University in Toruń, L. Rydygier Collegium Medicum in Bydgoszcz, 9 M. Skłodowska-Curie St., 85-094 Bydgoszcz, Poland; 2 Department of Clinical Microbiology, Antoni Jurasz University Hospital No. 1, Bydgoszcz, 9 M. Skłodowska-Curie St., 85-094 Bydgoszcz, Poland

**Keywords:** antimicrobial resistant, ascorbic acid, biofilm formation, *Proteus mirabilis*

## Abstract

Vitamin C has antimicrobial activity and is often used as an oral supplement accompanying antibiotic treatment in urinary tract infections (UTI). *Proteus mirabilis* is the third common species responsible for UTIs that are mostly treated with fluoroquinolones or aminoglycosides. Treatment of the UTI caused by *P. mirabilis* is problematic due to the ability to form biofilm on the urinary catheters. The aim of the study was to evaluate the influence of ascorbic acid in combination with antibiotics on *P. mirabilis* abilities to form biofilm. The susceptibility of *P. mirabilis* reference strain ATCC^®^ 29906™ and four clinical strains isolated from the urine samples of patients with urinary catheter were evaluated according to EUCAST recommendations. The influence of ascorbic acid (0.4 mg × mL^−1^) in combination with antibiotics on biofilm formation was evaluated spectrophotometrically. Aminoglycosides at sub-inhibitory concentrations more successfully limited biofilm formation by *P. mirabilis* strains without ascorbic acid addition. Inhibition rate differences at the lowest concentrations of gentamicin and amikacin were statistically significant (*p* ≤ 0.05). Ascorbic acid addition to the culture medium limited the inhibitory effect of fluoroquinolones, facilitating biofilm formation by *P. mirabilis* strains. The addition of ascorbic acid during aminoglycosides therapy may disturb treatment of urinary tract infections related to the presence of *P. mirabilis* biofilm.

## 1. Introduction

Vitamin C (ascorbic acid, AA) was first identified in the 1920s by a Hungarian biochemist, Albert Szent-Györgyi [1]. This vitamin cannot be synthesized by humans, therefore dietary supplementation of about 75–1000 mg per day is needed [1]. Ascorbic acid (after oral intake) is absorbed from the small intestine and transported into cells by specific transporters: Sodium-depend Vitamin C Transporters (SVCTs)-1 or -2. After absorption, vitamin C is distributed by blood to the tissues, especially the liver, brain, and adrenals. The concentration of AA in human tissues (200–2300 µM, depending on the organ) is much higher than in body fluids (i.e., 50–70 µM in plasma) and depends strongly on dietary intake of vitamin C [2]. Daily intake of AA impacts also on its bioavailability—it is very high if AA is supplemented in small doses (15–30 mg/day) and decreases if high doses are provided [2]. Higher doses (>100 mg/day) increase plasma AA concentration, and are almost totally excreted in the urine [3]. The urine excretion of vitamin C is much higher in humans taking a dose of 2000 mg, when compared to 100 mg of AA per day [1,4].

Vitamin C has antioxidant properties, therefore any effects of its presence in the human body may be most prominent under conditions of the enhanced oxidative stress, i.e., during the process of inflammation [5]. Viral, bacterial or fungal infections cause reactive oxygen species (ROS) release by phagocytes. It is helpful in the limitation of infection through deactivation of viruses or bacteria killing. ROS may also cause damage to the host cells, therefore the level of ROS released by phagocytes should be reduced directly after infection [6]. Vitamin C is an essential co-enzyme in the oxidative stress pathways, capable of ROS removal [5].

This vitamin C has also direct antimicrobial activity [7]. The growth of strains belonging to the two most common bacterial uropathogens (*Escherichia coli* and *Klebsiella pneumoniae*) is inhibited at the vitamin C concentration of 10 mg × mL^−1^ and 20 mg × mL^−1^ [7].

In general, vitamins may alter antimicrobial activity of particular antibiotics [8]. Carlsson et al. (2005) [9] and Afzal et al. (2017) [10] showed an increased antimicrobial activity of fluoroquinolones and aminoglycosides during AA addition. Habash et al. (1999) [11] additionally proved that vitamin C decreases the adhesion and microorganisms colonization of the biomaterials used in diagnostic/treatment procedures involving the urinary tract. Bacteria derived from the biofilm structure formed on the biomaterials are strongly related with catheter-associated urinary tract infections (CAUTIs).

According to the literature, CAUTIs belong to one of the most frequent nosocomial infections that may result in increased mortality and prolong hospitalization time and their costs [12,13,14].

*P. mirabilis* is the third common (after *E. coli* and *K. pneumoniae*) species responsible for urinary tract infections (UTIs) and the second cause of the CAUTIs [15,16]. Bacteria of *Proteus* genus are Gram-negative motile rods, belonging to the *Morganellaceae* family [17]. The most important representative of the genus is *P. mirabilis* [18]. Depending on the environment conditions, *P. mirabilis* strains may form biofilm with typical nutritional channels or a rather plain structure without those channels but with characteristic swarming cells fraction [19]. *P. mirabilis* rods might form biofilm on the surfaces of the different polymers that the catheters are made from [20]. The most important therapeutic obstacle connected with biofilm-submerged cells is their changed susceptibility to antibiotics, compared to the cells of planktonic form. Biofilm resistance to antibiotics may result from different mechanisms, frequently co-existing in the same bacteria cell population [21].

Due to the presence of vitamin C in urine during dietary supplementation, often accompanying antibiotic treatment in urinary tract infections, we examined minimal inhibitory concentration (MIC) values of AA for *P. mirabilis* strains, and the impact of AA at the concentration obtained after oral supplementation of vitamin C (0.4 mg × mL^−1^) with aminoglycosides and fluoroquinolones antibiotics on biofilm formation by antimicrobial-sensitive *P. mirabilis* strains.

## 2. Results

### 2.1. Minimal Inhibitory Concentration of Antibiotics and Ascorbic Acid

All the strains included in the study were confirmed for the sensitivity to the used antibiotics. MIC values of the antibiotics and AA for each particular strain are set in Table 1. 

### 2.2. Biofilm Formation

**Figure 1:**
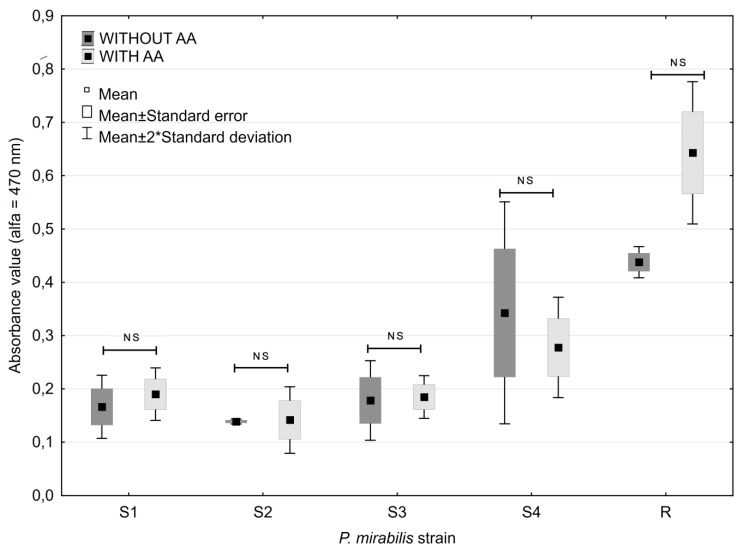
Absorbance values corresponding to biofilm formation in the presence of ascorbic acid (0.4 mg × mL^−1^).

All the studied strains were able to form biofilm on the surface of polystyrene plates (Figure 1). The highest absorbance values of formazan were obtained for the R strain—the average value of absorbance was 0.4377. Amongst the clinical strains, the highest absorbance was found for the S4 strain (0.3427). That strain was isolated from the youngest patient (18 months old) without current antimicrobial therapy. 

### 2.3. Ascorbic Acid Impact on Biofilm Formation

Generally, AA at the concentration of 0.4 mg × mL^−1^ did not change biofilm formation by *P. mirabilis* strains. The highest observed alteration was achieved for the R strain. The average absorbance values measured for the biofilm of *P. mirabilis* R strain in the medium without AA were 0.438, while the corresponding value for the AA-supplemented medium were generally higher, 0.643 (Figure 1). However, the differences were not statistically significant (*p* > 0.05).

### 2.4. Antibiotic Impact on Biofilm Formation

**Figure 2:**
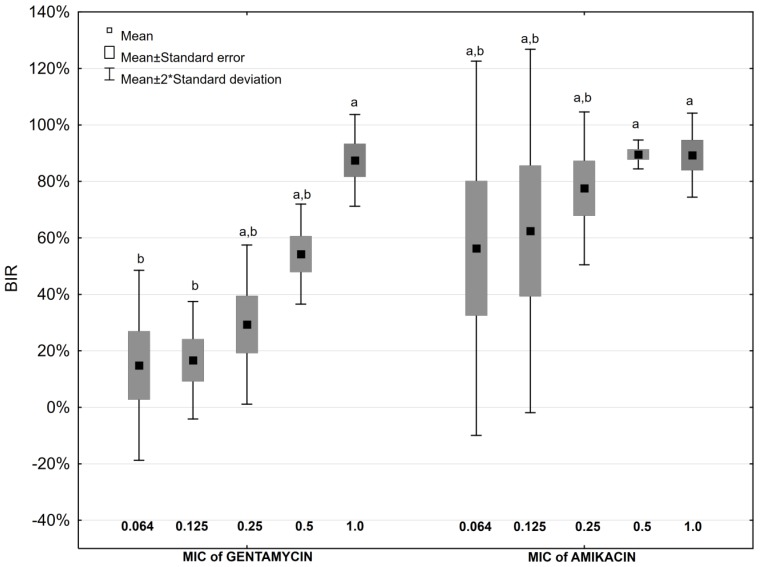
Biofilm Inhibitory Rate values in the presence of aminoglycosides (a,b,c,…—values marked with different letters differ statistically significant at the significance level *p* ≤ 0.05).

**Figure 3:**
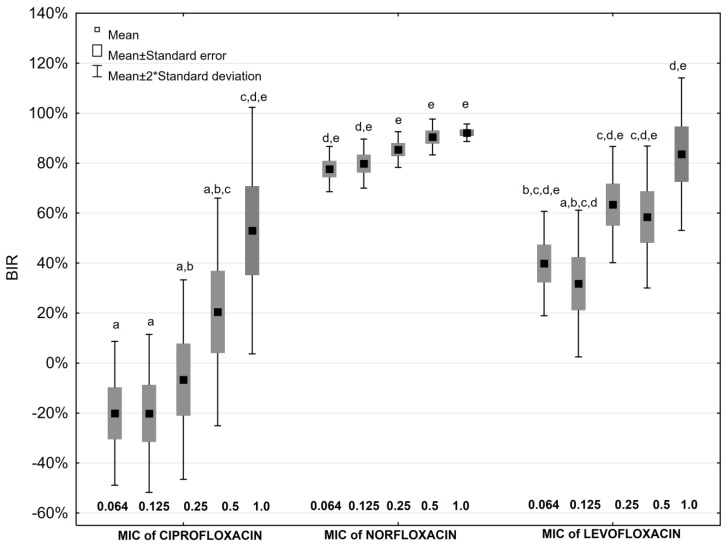
Biofilm Inhibitory Rate values in the presence of fluoroquinolones (a,b,c,…—values marked with different letters differ statistically significant at the significance level *p* ≤ 0.05).

All examined concentrations of aminoglycosides were able to inhibit *P. mirabilis* biofilm formation. The highest values of the biofilm inhibitory rate (BIR) were observed at concentrations of studied antimicrobials corresponding to 1 MIC measured for planktonic cells (Figure 2 and Figure 3). Amikacin at the concentration corresponding to 0.5 MIC and 1 MIC, and 1 MIC gentamicin were able to inhibit biofilm formation stronger than 0.064 MIC and 0.125 MIC of gentamicin (Figure 2).

Amongst fluoroquinolones the highest values of BIR at all the examined concentrations of antimicrobials were obtained for norfloxacin (Figure 3). All studied concentrations of norfloxacin inhibited biofilm formation stronger than any sub-inhibitory concentration of ciprofloxacin (Figure 3). Favourable *P. mirabilis* biofilm formation at 0.064 and 0.25 MIC of ciprofloxacin was also noticed.

### 2.5. Antibiotic and Ascorbic Acid Impact on Biofilm Formation

Aminoglycosides limited *P. mirabilis* strains biofilm formation more successfully in the medium lacking AA addition (Table 2). The differences in BIR values for both, gentamicin at the highest concentrations applied and amikacin at all studied concentrations, were statistically significant (*p* ≤ 0.05). Surprisingly, simultaneous supplementation of the culture medium with AA and the aminoglycosides at concentrations equal to 0.064 MIC and 0.125 MIC increases *P. mirabilis* biofilm production, while biofilm formation is limited in the presence of the antibiotic itself (Table 2).

Similar results were observed for fluoroquinolones—supplementation of the culture medium with 0.4 mg × mL^−1^ AA decreases inhibitory activity of these antimicrobials, facilitating *P. mirabilis* biofilm formation. The exceptions were the ciprofloxacin concentration of 0.064–0.25 MIC value and levofloxacin concentration of 0.064–0.125 MIC value, where BIRs were higher in medium with AA than without, but the differences were not statistically significant. The presence of AA with 0.25 MIC and 0.5 MIC of norfloxacin decreased BIR. The differences observed, were statistically significant (*p* < 0.05).

## 3. Discussion

*Proteus* spp. biofilm synthesis is a significant clinical problem in terms of its eradication need and patients’ treatment applied. These bacteria are able to synthesize extracellular structures on the surfaces of all known types of catheters [22]. Biofilm constitution limits antimicrobial penetration and distribution through the whole structure, mostly to the bacteria submerged in its deeper layers, generating sub-inhibitory antibiotics concentrations. Moreover, biofilm-associated microorganisms express higher resistance levels to antimicrobials, resulting in bacteria multiplying although the drug is present in the environment [23]. That is the reason why antimicrobials concentrations active against planktonic forms of bacteria, frequently are unsuccessful for biofilms. They remain at too low concentration or for too short time to penetrate successfully biofilm structure. In general, with increasing antibiotic concentration its inhibitory influence on bacterial biofilm grows [24,25]. Similar conclusions were made in our study. Amongst the examined antibiotics that are most commonly used in urinary tract infection treatment, norfloxacin demonstrates the most effective antibiotic amongst those detected at the sub-inhibitory concentrations. We observed than even the highest studied concentration of antimicrobials capable of inhibiting the growth of planktonic forms of bacteria were unable to inhibit biofilm formation. It may cause a failure in the antimicrobial treatment applied. That is why searching for the alternative therapeutic solutions, disrupting antimicrobial biofilm activity at lower concentrations, is fully understood.

We found that 10 mg × mL^−1^ of AA inhibit growth of *P. mirabilis* planktonic cells. That confirms the results obtained by Verghese et al. (2017) [7]. They demonstrated antibacterial activity of 10 mg × mL^−1^ vitamin C on *E. coli* and *K. pneumoniae* strains isolated from urine samples. This activity is dose-depend and independent of the antimicrobial susceptibility patterns of the strains. Concentration of AA in urine in the bladder after oral-supplementation of vitamin C is much lower [4], therefore we examined the impact of a lower concentration of AA. In this study we found that 0.4 mg × mL^−1^ of AA, which is possible to obtain in the bladder, does not inhibit biofilm formation by *P. mirabilis* strains.

Similar results were obtained by Carlsson et al. (2005) [9]. They examined the influence of 10 mM AA solution on two *E. coli* reference strains growth on the surface of the urinary catheter and they noted 1000-fold (10^5^ to 10^8^) increase in the number of both bacteria strains after application of one vitamin C dosage to the culture medium for 24 h. El-Gebaly et al. (2012) [24] observed conflicting results: 65–78% and 85–90% biofilm formation inhibition while 80 and 100 mg × mL^−1^ of vitamin C were used, respectively. The concentrations of AA applied by them [5] were 8 and 10-fold higher than MIC values of vitamin C noted for the planktonic form of clinical *P. mirabilis* strains examined in our study, so it is very probably that concentrations higher than MIC values are able to inhibit biofilm formation. Habash et al. (1999) [11] showed that the presence of AA in the urine of patients inhibits *E. coli* and *Enterococcus faecalis* adhesion to biomaterials that the catheters are made from. This tendency was not observed for other urinary tract pathogens [11].

The strategy of our own study presented was to evaluate the influence of AA addition on antibiotics activity against *P. mirabilis* biofilm. Vitamin C is commonly prescribed with antibiotics during UTI treatment [26]. In our research, AA addition resulted in decreasing fluoroquinolones and aminoglycosides inhibitory effect on *P. mirabilis* strains biofilm formation. Similar results for the planktonic cells were obtained by Masadeh et al. (2012) [26] for ciprofloxacin. Pre-treatment of *E. coli* cells with 10 mM AA inhibits ciprofloxacin-induced hydrogen peroxide generation and reverse its antibacterial activity. Moreover, Aiassa et al. (2012) [27] investigated the decreased activity of ciprofloxacin on *P. mirabilis* strains when applied simultaneously with 10 mM of vitamin C. The observed decreased sensitivity to ciprofloxacin was explained by antioxidant activity of vitamin C, influencing the ciprofloxacin mode of action on bacteria cells, based on oxidative stress induction. The increase of MIC value for another fluoroquinolone (pefloxacin) was also detected for *S. aureus* in the presence of vitamin C in the study conducted by Awofisayo et al. (2012) [28].

The conflicting conclusions were obtained for higher concentrations of vitamin C (80 and 100 mg × mL^−1^) applied on biofilm examination by El-Gebaly et al. (2012) [24]. They detected synergistic activity of levofloxacin and vitamin C in biofilm reducing activity on the surface of the urinary catheter for various uropathogens, including *P. mirabilis*. The planktonic forms of both strains examined by them [24] were resistant to ciprofloxacin; while in the study presented, strains were sensitive to all the antibiotics applied. This, and 10-fold higher than MIC of AA concentrations used in the study, might have resulted in obtaining different results. Pandit et al. (2017) [29] demonstrated that low concentrations of vitamin C (10–30 mM of sodium ascorbate) also inhibits exopolysaccharide of *Bacillus subtilis* biofilms synthesis may led to the increase of bacteria susceptible to vitamin C-induced oxidative stress compounds or antimicrobials. Yassein (2004) [30] explains that higher activity of levofloxacin observed after environment pH growth by AA is more optimal level for this fluoroquinolone activity. Moreover, vitamin C plays a crucial role in the quorum sensing phenomenon inhibiting competitively autoinducer AI-2 [31].

The influence of antioxidants on aminoglycosides remains unknown. However, Cursino et al. (2005) [32] demonstrated a synergic effect of kanamycin or streptomycin and 1.0 mg × mL^−1^ of AA against multidrug resistance *P. aeruginosa* strains, but they did not find any interactions between tobramycin and the same concentration of AA. Goswami et al. (2007) [33] observed that AA and glutathione, both at the concentrations of 10 mM, decreased the sensitivity of the *E. coli* strain to streptomycin in a manner similar to fluoroquinolones. Andrade et al. (2014) [34] study obtained that antioxidants—alpha-tocopherol enhances the antibiotic activity of aminoglycosides against Gram-negative bacteria, which have a higher level of antimicrobial resistance. Shahzad et al. (2018) [8] also observed the synergistic effect of water-soluble and fat-soluble vitamins, including vitamin C, with beta-lactams against *Acinetobacter baumannii* and methicillin-resistant *S. aureus* (MRSA). They do not observed this effect for any of the examined aminoglycosides. In this study we demonstrated that AA strongly decreases the BIR value. Moreover, we observed the enhancement biofilm formation at the lowest sub-inhibitory concentrations of amikacin or gentamicin in the presence of AA while in the medium supplemented only with the antimicrobials—the inhibition of biofilm formation was demonstrated. Similar results were also proven by Wasfi et al. (2012) [25] for ceftriaxone applied at the concentration equal to 0.125 of MIC for this cephalosporine.

In the available literature, very few information on the influence of vitamin C supplementation on the CAUTIs treatment can be found. Thus, further studies of that issue are necessary, especially in terms of bacterial biofilm production.

## 4. Materials and Methods

### 4.1. Strains Origin

*Proteus* spp. strains were isolated from patients of the Antoni Jurasz University Hospital No 1 in Bydgoszcz, Poland. The examination included only strains obtained from urine samples collected using bladder catheters. Strains were identified during microbiological investigation at the Department of Microbiology.

The susceptibility of *Proteus* spp. strains was examined using BD Phoenix™ system (Becton Dickinson) and interpreted according to EUCAST recommendation [35]. Four randomly chosen clinical strain (named S1−S4), susceptible to the examined antimicrobials and one reference strain (R) purchased from American Type Culture Collection (ATCC^®^ 29906™) were included into the study (Table 3). Strains identification was confirmed using MALDI-TOF MS technique (Microflex, Bruker). Investigation strains were stored in a Brain-Heart Infusion (BHI, Becton Dickinson) with 20.0% glycerol (Avantor) at −70 °C.

### 4.2. Minimal Inhibitory Concentration of Antibiotics and Ascorbic Acid

The MIC value for gentamicin (GEN), amikacin (AMK), ciprofloxacin (CIP), norfloxacin (NOR), levofloxacin (LEV), and ascorbic acid (AA) (all delivered by Sigma Aldrich) of *P. mirabilis* strains was examined by the microdilution method in the microtiter plates method according to the Clinical and Laboratory Standards Institute (CLSI) recommendation (2012) [36]. The growth of bacteria in the presence of examined antibiotic and AA at concentrations ranging from 0.001–128.0 μg × mL^−1^ was evaluated. *Escherichia coli* strain ATCC^®^ 25922™ was used for the control assays for antimicrobials. The MIC value was read-out visually by the presence of turbidity of the suspension in the wells of microtiter plates at a given concentration of antibiotic or AA. Investigation was done in three independent replications.

### 4.3. Biofilm Formation

The ability to form biofilm by the studied strains was examined using 0.1% 2,3,5 triphenyltetrazolium chloride (TTC, Sigma Aldrich) by the microtiter methodology that was described previously [37]. That colorless substance is metabolized by living cells to red product-formazan. The investigation was done in three independent replications. Absorbance (A) read-outs were conducted with a spectrophotometer at the wavelength of 470 nm using KC4™ v3.4 and KC4™ Signature programs (BioTek).

### 4.4. Ascorbic Acid Impact on Biofilm Formation

The examined strains of *P. mirabilis* were cultivated on the Mueller-Hinton Agar II (MHA, Becton Dickinson) and cultured at 37 °C for 18 h. Next, the single colonies of each strain were inoculated into Tryptic Soy Bullion (TSB, Bio-Rad). After 18 h at 37 °C, each culture was centrifuged for 15 min at 4000 rpm and the supernatants was discarded. The remaining pellet was rinsed with 3 mL of phosphate buffered saline solution (pH = 7.2) (PBS, Avantor). Next, the bacterial suspension was centrifuged at 4000 rpm for 10 min and the pellet was used to make the suspension of 0.5 McFarland turbidity in Mueller-Hinton Broth (MHB, Becton Dickinson). Then, 100 μL of every suspension was placed in the wells of polystyrene 96-well plate, three repetitions each. The wells were filled with: (a) 100 μL of sterile MHB (biofilm formation evaluation/positive control), (b) 100 μL of AA (to the final concentration of 0.4 mg × mL^−1^) in MHB (AA impact assessment). The culture was incubated in a humid chamber at 35 ± 2 °C for 24 h. After incubation, the solutions were removed and the wells rinsed tree times with sterile PBS. Next, 100 μL of TSB and 100 µL of 0.1% TTC were added to each well. The plates were placed on a shaker at 250 rpm for 5 min at room temperature. Next, the plates were placed at 37 °C. After 3-h incubation, the TTC was removed and plates were rinsed three times with sterile water. Finally, 200 μL of 96% methanol (Avantor) were added to each well and left on a shaker at 400 rpm for 5 min at room temperature. Results were conducted with a spectrophotometer as described above.

### 4.5. Antibiotic Impact on Biofilm Formation

Based on MIC values results, the sub-inhibitory concentration of the studied antibiotics were prepared. The antibiotic concentrations were equivalent to 0.064, 0.125, 0.25, 0.5, and 1.0 of MIC values for particular antimicrobial. The preparation of examined strains inoculum was based on methods described in the previous section. Bacteria suspension (100 μL) was placed into the wells of polystyrene 96-well plate. The wells were filled with: (a) 100 μL of an antimicrobial at the concentration in final medium in range 0.064–1 MIC, diluted in MHB (sub-inhibitory antibiotic concentration impact assessment), (b) 100 μL of MHB only (biofilm formation evaluation/positive control). The culture of biofilm was conducted under conditions described above. Results were given as absorbance values.

### 4.6. Antibiotic and Ascorbic Acid Impact on Biofilm Formation

Preparation of the examined strains inoculum based on the methods was described in the previous section. Bacteria suspension (100 μL) was placed in the wells of polystyrene 96-well plate. Then, the wells were filled with: (a) 50 μL of an antibiotic at concentration in the final medium in the range 0.064–1 MIC, diluted in MHB, and 50 μL of sterile MHB (antibiotic impact evaluation), (b) 50 μL of an antibiotic at the same concentration as in the previous point, diluted in MHB, and 50 μL of AA (to final concentration 0.4 mg × mL^−1^) in MHB (antibiotic + AA impact estimation). The culture of biofilm was conducted under conditions described above. Results were given as absorbance values.

### 4.7. Biofilm Inhibition Rate

We introduced value described as Biofilm Inhibition Rate (BIR) to express our final results. It normalized the impact of TTC to formazan metabolism measurement time, regardless of the examined strain properties. BIR is based on absorbance values and is calculated according to the formula:

(1) $$\mathrm{BIR}=\frac{Ax-Ay}{Ax}*100\%$$

where (*Ax*) stands for the absorbance value of positive control, and (*Ay*) for the absorbance value of the strain subjected defined concentration of antibiotics or/and ascorbic acid.

### 4.8. Statistical Analysis

Statistical analysis was performed using Statistica 12.5 PL (StatSoft) software. The confidence interval for all the tests applied was α = 0.05. The obtained results normality was evaluated with the Shapiro-Wilk test. Bonferroni test was applied to compare the impact of various concentrations of the studied antibiotics on biofilm formation (Figure 2, Figure 3), and U Mann-Whitney test was used to compare results obtained for AA (Figure 1), and AA with and without antibiotics (Table 2).

## Figures and Tables

**Table 1:** The Minimal Inhibitory Concentration values of the examined antibiotics and ascorbic acid.

Strain No.	GEN [µg × mL^−1^]	AMK [µg × mL^−1^]	CIP [µg × mL^−1^]	NOR [µg × mL^−1^]	LEV [µg × mL^−1^]	AA [µg × mL^−1^]
S1	0.500	0.016	0.001	0.004	0.016	10.000
S2	0.250	0.016	0.001	0.002	0.008	10.000
S3	0.004	0.002	0.001	0.002	0.008	10.000
S4	0.500	0.008	0.001	0.008	0.016	10.000
R	0.250	0.016	0.001	0.004	0.016	10.000

GEN—gentamicin, AMK—amikacin, CIP—ciprofloxacin, NOR—norfloxacin, LEV—levofloxacin, AA—ascorbic acid, S1-4—clinical strains, R—reference strain.

**Table 2:** *Proteus mirabilis* average Biofilm Inhibition Rate in terms of antibiotic MIC values with (+AA) and without ascorbic acid addition (0.4 mg × mL^−1^).

Antibiotic Concentration	Biofilm Inhibition Rate [%]														
	Aminoglycosides						Fluoroquinolones								
	GEN	*p* *	GEN + AA	AMK	*p* *	AMK + AA	CIP	*p* *	CIP + AA	NOR	*p* *	NOR + AA	LEV	*p* *	LEV+AA
0.064 MIC	14.9	NS	−94.7	56.3	0.028	−63.7	−20.1	NS	−9.5	77.6	NS	68.4	39.8	NS	40.6
0.125 MIC	16.7	NS	−50.5	62.5	0.047	−6.0	−20.2	NS	−11.1	79.8	NS	77.4	31.8	NS	42.3
0.25 MIC	29.3	NS	0.7	77.6	0.016	9.5	−6.6	NS	−5.8	85.4	0.047	73.1	63.4	NS	50.5
0.5 MIC	54.3	NS	5.6	89.4	0.009	8.8	20.5	NS	7.6	90.4	0.047	75.7	58.5	NS	48.3
1 MIC	87.5	0.028	41.6	89.3	0.028	54.3	53.0	NS	32.8	92.2	NS	91.1	83.6	NS	66.5

GEN—gentamicin, AMK—amikacin, CIP—ciprofloxacin, NOR—norfloxacin, LEV—levofloxacin, * *p*-value—statistically significant differences in the level of biofilm inhibition depending on the addition of ascorbic acid.

**Table 3:** Characteristics of *P. mirabilis* clinical isolates.

Strain No	Disease Diagnosis	Sex	Patients Age	Antibiotic Treatment
S1	UTI	Female	85 Years	Amoxicillin/clavulanic acid
S2	Abdominal hernia surgery	Female	59 Years	None
S3	UTI (urinary stones)	Female	86 Years	Ciprofloxacin
S4	UTI	Male	18 Months	None

UTI—Urinary Tract Infection.
